# The role of gender in the association between personality and task priority in older adults’ dual-tasking while walking

**DOI:** 10.1186/s12877-017-0691-1

**Published:** 2018-01-02

**Authors:** Maayan Agmon, Galit Armon, Shani Denesh, Mihalis Doumas

**Affiliations:** 1 Department of Nursing, Faculty of Social Welfare and Health Sciences, University of Haifa, 31905 Haifa, Israel; 2 Department of Psychology, University of Haifa, 31905 Haifa, Israel; 3 School of Psychology, Queen’s University Belfast, 18–30 Malone Road, Belfast, BT9 5BN UK

**Keywords:** Personality, Dual-task, Older adults, Dual-task cost, Extraversion, Conscientiousness

## Abstract

**Background:**

Falls are a major problem for older adults. Many falls occur when a person’s attention is divided between two tasks, such as a dual task (DT) involving walking. Most recently, the role of personality in walking performance was addressed; however, its association with DT performance remains to be determined.

**Methods:**

This cross-sectional study of 73 older, community-dwelling adults explores the association between personality and DT walking and the role of gender in this relationship. Personality was evaluated using the five-factor model. Single-task (ST) and DT assessment of walking-cognitive DT performance comprised a 1-min walking task and an arithmetic task performed separately (ST) and concurrently (DT). Dual-task costs (DTCs), reflecting the proportional difference between ST and DT performance, were also calculated.

**Results:**

Gender plays a role in the relationship between personality and DT. Extraversion was negatively associated with DTC-motor for men (Δ*R*^2^ = 0.06, *p* < 0.05). Conscientiousness was positively associated with DTC-cognition for women (Δ*R*^2^ = 0.08, *p* < 0.01).

**Conclusion:**

These findings may lead to effective personality-based early detection and intervention for fall prevention.

## Background

Advanced age is often accompanied by mobility limitations [1]. Over 35% of adults age 70 and above, and the majority of those age 85 and above, have clinically diagnosable gait abnormalities [1]. Older adults who experience gait abnormalities often rely more on executive than on automatic control of walking, and this greater reliance on executive control has been associated with increased risk of falls [2, 3]. The most common approach to extracting insights about executive versus automatic control of walking is the dual-task (DT) paradigm [3]. Indeed, most everyday situations that involve walking—walking while talking on the phone, or crossing the street while paying attention to a complex scene—require performing two tasks simultaneously. Efficient allocation of attention between two tasks is critical to maintaining safety during walking and to reducing fall accidents [4]. However, often older adults divide their attention inefficiently between a task involving balance, or postural control, and a cognitive task. Instead of prioritizing the postural task and choosing the “posture first strategy,” they opt for the “cognitive first strategy” and compromise their safety [5, 6]. Thus, evaluating task prioritization is critical to illuminating older adults’ DT performance.

DT interference occurs when there are competing demands for attentional resources [7]. Specifically, when the attentional demands of the two tasks exceed the total attention capacity, performance of the motor task, the cognitive task, or both may decline relative to single-task (ST) performance [3]. Wollesen et al. [8] reviewed theories that explain how people divide attention during different DT situations, that is, the difference between ST and DT performance, or proportional DT costs (DTCs). The main theories they discussed are the limited resources hypothesis, the cross domain model, the supra postural task model, and the prioritization model. Their main claim is that each can explain different DT combinations and that no single model fits all task combinations. For example, higher DTCs in the motor task, which are often accompanied by prioritization of the cognitive task, are associated with decreased walking automaticity and an increased risk of falls in older adults [3, 8, 9]. Most studies that explore DT performance in older adults evaluate either cognitive or motor performance, without considering DTCs for both tasks [10]. Such an approach does not provide the information necessary to understand resource allocation dynamics between the tasks [11] or to draw a comprehensive picture of attentional resource allocation in two tasks [3].

Previous studies have identified a number of factors affecting the decreased automaticity in walking tasks and the general DT impairment observed in older adults. The main factors studied were physiological age-related changes [3, 12] including nervous system damage, proprioception, touch, pain, cognition, biomechanical constraints, and hearing. Most of these factors are associated with the aging process itself and develop late in life. Most recently, however, two studies emphasize the need to further explore individual differences, personal traits, and their influences on walking deterioration over the lifespan [10, 12]. The ability to DT begins early in life and develops over the lifespan [10]. Thus, DT research should take into account factors that may influence DT through the lifespan and not only aging-associated factors [6, 10]. Personality has been linked to both the risk of developing age-related disabilities and to longevity [13], as well as to walking deterioration [14]. Moreover, psychological theories have linked fear of falling and anxiety to falls [15]. The link between anxiety and personality is well established [16, 17]. However, the contribution of personality to DT walking deterioration and prioritization with aging, which increases risk of falls, remains to be explored.

In health research, personality is often assessed by means of the five-factor model (FFM, also known as the Big Five) [13, 18]. The FFM classifies most personality traits under one of five dimensions: Extraversion, Agreeableness, Conscientiousness, Neuroticism, and Openness to experience [18]. Personality has been linked to many health outcomes that are relevant for older adults, including motor function [19], gait speed [20] and muscle strength [21], mortality [22], and morbidity [23] and mobility [24]. Several specific pathways may explain relationships between personality and deterioration in DT walking. Cognitive pathways suggest that high Openness and high Conscientiousness are associated with slower rates of cognitive decline in aging (for a review, see Curtis et al. [25]). Behavioral pathways suggest that some personality factors, such as low Conscientiousness and high Neuroticism, are strong predictors of unhealthy behaviors, including physical inactivity and excess body weight, that result in limited walking abilities [26]. Another indirect pathway links personality type and the increased likelihood of developing chronic or acute illnesses, which in turn might initiate a process that leads to mobility dysfunction or that speeds its progression [27]. Thus, the link between personality and aging, longevity, and morbidity is well established. However, the link between personality and mobility decline in older adults is rarely explored.

Although the topic has scarcely been studied, few personality traits have been associated with mobility performance, and the findings are discrepant. For example, higher levels of Extraversion and Conscientiousness were found to be associated with reduced risk of disability with aging [28]. Another study emphasized the contribution of higher Openness in protecting against mobility deterioration [20]. These studies relied on self-reporting of mobility performance. Yet, even with more objective measures, findings are ambivalent. Tolea and colleagues demonstrate that higher Conscientiousness, but not Openness, is associated with faster initial gait speed and less decline over a 3-year period. Also, people characterized by high Neuroticism, either in isolation or in combination with low Extraversion and low Conscientiousness, are more prone to having low muscle strength [21]. Most recently, LeMonda et al. [29] showed for the first time that older adults with high Neuroticism and low Extraversion demonstrate greater DTC for both the cognitive and the motor task during DT walking when compared to other combinations of Extraversion and Neuroticism. This study paved the way for the establishment of the relationships between personality and the ability to divide attention while walking and performing another task. However, several questions remain about the roles of the other three FFM traits in relation to DT and personality, and about whether gender plays a role in this association.

Research on the association between personality and mobility is in its infancy. The evidence is mixed, and an exploration of functional mobility that represents the relationship between the full spectrum of personality factors (as modeled by the FFM) and DT walking has yet to occur. Expanding our understanding of this relationship may contribute to developing new, tailored fall-prevention strategies for elderly adults by enabling early detection of people at risk based on personality type. Thus, the objective of the current study is to explore the association between personality and DT walking performance in community-dwelling older adults. Based on our previous investigation [24], we hypothesize that people with higher Extraversion and higher Conscientiousness perform DT better and thus have lower DTC.

Following the body of evidence on gender differences in DT gait performance [30, 31] and in the levels of the FFM [32], we also investigated gender differences in the association between the FFM and DT prioritization, in addition to using gender as a control variable. However, because no past study focused on gender differences in the effects of the FFM on DTC, we tested these gender differences on an exploratory basis only.

## Methods

### Sample

To calculate the sample size, we used G-Power analysis software [33] and considered an OLS regression model with five independent variables, six covariates and two interactions; defining a medium effect size (f2 = 0.15) of α = 0.05, a power of .80 required a total sample of 78 participants.

### Participants

In this cross-sectional study, 90 participants were recruited (55 female and 35 male), and 10 did not meet the inclusion criteria (for five Hebrew was not the first language, two reported back pain during walking and three did not complete the protocol). We have full data for 73 community-dwelling older adults (mean age 75 years, SD 6.0, 35 males and 38 females) recruited through advertisements in their communities. Inclusion criteria were being age 65 or older, being able to walk independently, and being able to speak, understand and read Hebrew. The study procedures were approved by the University of Haifa’s Institutional Review Board.

### Tasks and procedures

Data were collected in community centers by one investigator (SD). Data collection sessions lasted 90 min and included collection of demographic information about participants’ age, gender, disease burden (the presence of neurologic or musculoskeletal diagnosis such as cerebral vascular accident, Parkinson disease, Alzheimer disease or multiple sclerosis; severe orthopedic limitations such as acute back pain or a total hip or knee replacement; significant hearing or vision loss not corrected with hearing aids or glasses), and number of weekly hours customarily engaged in physical activity. Cognitive ability was assessed with the MoCA, which covers 10 cognitive domains using rapid, sensitive, and easy-to-administer cognitive tasks. MoCA sensitivity to detect minimal cognitive impairment (MCI) is 100%, and specificity is 87% [34]. Personality was assessed using the NEO-FFM, a short version of the original FFM [18]. It includes 60 statements on five domains of personality: Neuroticism, Extraversion, Openness, Agreeableness, and Conscientiousness, rated using a five-item Likert scale ranging from 1 (*extremely inaccurate*) to 5 (*extremely accurate*). Internal consistency ranges from α = 0.70 to α = 0.86, and construct validity correlations with the original FFM range from *r* = 0.76 to *r* = 0.91 [18].

Participants were asked to perform the following three tasks in random order. Each task lasts 1 min. (a) ST 1-min walk, during which participants walked back and forth on a 10-m course. The total distance walked was recorded. (b) ST subtraction by 3, starting from a random number from 100 to 250. The number of correct answers was calculated. This task was conducted while the participant was seated. (c) DT performance of (a) and (b). This task is often used for cognitive evaluation alone, or concurrently with a motor task [35]. The number of correct answers and the distance walked were calculated. During DT performance, participants were instructed to conduct each task to their best ability.

DTCs for walking and for the cognitive task were calculated for each test. DTCs reflect the cost of DT performance compared to ST performance and are expressed as a percentage of ST performance: DTC = ([DT – ST] / ST) × 100 [36]. DTCs for the walking task are lower in young adults than in older adults and are strongly associated with an increased risk of falls in the elderly.

### Statistical analyses

SPSS 19 software was used for the following analyses: (1) to perform *t* tests contrasting ST with DT performance, (2) to assess whether DTCs were significantly different from zero, and (3) to calculate ordinary least squares (OLS) regressions to examine the associations between personality factors and the ability to divide attention between the two tasks of walking and subtracting by 3. In the first step of the regression analysis, the main effect variables of the FFM traits were entered simultaneously, to isolate the unique effect of each domain. In the second step of the regression, potential covariates of age, gender, MoCA, body weight, physical activity and chronic disease were entered. These covariates were considered potential mediators of personality associations with DTC performance. Attenuation attributed to the possible mediators under consideration was calculated using the formula 100 × (β Model 1 − β Model 2) / (β Model 1) (e.g. Hagger-Johnson et al. [37]). In the third step, because of sex differences related to changes in both personality and mobility [38, 39], we tested gender as a moderator by using interactions of the FFM with gender. We used the centered values of the FFM in testing these interaction terms to reduce multicollinearity and to facilitate the interpretation of interactions if found in our data analyses. The regression models with the interactive associations also include the linear, centered terms. To reduce the possibility of multicollinearity among the interaction and quadratic terms and their component predictors, all predictors were centered prior to the regression runs [40]. In Table 1, only significant interactions are shown (Table 1). In order to complement the inferential statistics of *P*-values, the effect size of the main effects of the FFM was also calculated as the square of the Pearson correlation *r* (*R*^2^) to reflect the proportion of variance shared by the FFM and DTC [41].

**Table 1:** Participant characteristics by gender (*N* = 73)

Characteristic	Male mean ± SD	Female mean ± SD	*P*-value
Age, years	74.7 ± 6.5	73.9 ± 6.0	.738
Openness	3.31 ± .54	3.39 ± .47	.452
Neuroticism	2.52 ± .56	2.64 ± .47	.208
Agreeableness	3.69 ± .51	4.10 ± .46	.47
Conscientiousness	4.00 ± .61	3.80 ± .51	.143
Extroversion	3.23 ± .35	3.7 ± .43	.419
MoCA	22.12 ± 3.2	21.9 ± 2.9	.438
ST walk (meters)	52.93 ± 6.1	53.87 ± 7.8	.13
DT walk (meters)	41.3 ± 10.3	42.0 ± 4.9	.09

*MoCA* Montreal Cognitive Assessment, *ST* single task, *DT* dual task

## Results

A total of 81 community-dwelling, older adults, 35 males and 38 females, completed the study. Mean age was 75 years (SD 6.0) (Table 1). The mean BMI was 26.1 (SD 4.1), and the mean MoCA score was 23.0 (SD 3.1). Of the 73 participants, 13 (16.7%) reported having experienced at least one fall in the previous year. The mean distance walked during ST was 53.96 m (SD 11.6) and during DT was 41.83 m (SD 11.46). A significant difference was found between distance walked in ST and DT (*t* = 11.41, *df* = 87, *p* < 0.001). The mean number of correct answers in the ST cognitive task was 26.32 (SD 9.6) and in the DT was 23 (SD 9.62). A significant difference was found between ST and DT cognitive performance (*t* = 4.27, *df* = 87, *p* < 0.001). The mean relative difference between walking as a ST and as a DT (DTC) was 25.57% (SD 9.5) and for the cognitive tasks was 7.5% (SD 4.3). In addition, DTCs were significantly larger for walking (*t* = 3.1, *df* = 87, *p* = 0.003), suggesting that participants prioritize cognitive performance at the expense of walking.

Results of the OLS regression analyses model show that the association between the FFM and the ability to divide attention between two tasks while walking was not significant. The inclusion of age, MOCA, body weight, physical activity and chronic disease in the model did not change the associations between personality traits and DT; thus, no mediating effects of the covariates were found.

### Gender differences: exploratory analysis

We tested, on an exploratory basis, whether gender moderated the hypothesized associations between the FFM traits and DTC by interaction term. The results are reported in Table 2. We found that after the inclusion of the interactions, Extraversion was positively associated with DTC-motor (β = .39, *p* < 0.05) and that the interaction of Extraversion with gender was significant (β = −1.84, *p* < 0.05). The interaction was plotted according to Aiken et al. [42]. The plot (not shown) indicated that Extraversion was positively associated with DTC-motor for men only (β = .88, *p* < 0.05). The effect size, as based on the Δ*R*^2^ of the interactions of gender with Extraversion in the OLS regressions, was significant *(*Δ*R*^2^ = 0.07, *p* < 0.05). Additionally, we found a significant interaction between Conscientiousness and gender (β = 2.03, *p* < 0.01). Plotting this interaction (not shown) indicated that Conscientiousness was negatively associated with DTC-cognition for women only (β = −.32, *p* < 0.05). The effect size, as based on the Δ*R*^2^ of the interactions of gender with Conscientiousness, was significant (Δ*R*^2^ = 0.07, *p* < 0.05).

**Table 2:** Results of the OLS regression analyses testing the associations between the Five-Factor Model and the ability to divide attention between two tasks while walking

Variable	DTCmotor (*N* = 81)				DTCcog (*N* = 81)			
	*B*	SEB	β	95% CI	*B*	SEB	β	95% CI
Neuroticism	5.56	3.43	.20	−110.51-122.18	2.42	4.45	.07	−6.47-11.31
Extraversion	12.28*	6.14	.39	.02–24.53	3.52	4.84	.09	−6.14-13.17
Conscientiousness	2.42	4.05	.07	−5.66-10.50	−9.89	7.59	−.23	−25.04-5.25
Openness	−.35	4.54	−.01	−9.41-8.71	−.96	5.70	−.02	−12.33-10.41
Agreeableness	.06	4.58	.01	−9.08-9.21	−1.09	5.69	−.02	−12.46-10.27
*R* ^2^	.05				.03			
Co Covariates								
Age	.13	.38	.04	−.63–.89	−.196	.49	−.052	−1.18-.79
Gender	62.37**	25.02	1.75	12.45–112.29	−90.09*	41.52	−1.95	−172.95)--7.244(
MoCA	−1.06	.78	−.19	−2.62-.49	−.517	1.01	−.07	−2.54-1.51
Body weight	−1.12	.58	−.24	−2.28-.04	.390	.75	.06	−1.11-1.89(
Physical activity	−.67	.72	−.11	−2.10-.76	−1.976*	.96	−.25	−3.90-(−.04)
Chronic disease	.76	2.61	.03	−4.45-5.97	−1.775	3.42	−.06	−8.61-5.06
Δ*R*^2^		.06				.06		
Gender × Extraversion	−19.36*	7.94	−1.84	−35.20-(−3.510)		–		
Gender × Conscientiousness		–			23.973*	10.40	2.03	3.21–44.73
Δ*R*^2^		.07*				.07*		

*B* and β nonstandardized and standardized partial regression coefficients, respectively. *SEB* standard error of the former, *MoCA* Montreal Cognitive Assessment, *OLS* ordinary least squares; Gender (1 = women);

*p < 0.05; **p < 0.01

## Discussion

This study investigated whether prioritization strategies are associated with personality during walking in ecologically valid conditions (i.e. with DT) in community-dwelling older adults, controlling for cognitive ability, lifestyle habits, health status and gender. The association between personality and DT walking has rarely been explored. Previous studies have not considered all five traits of the FFM and have not accounted for the role of gender [29]. Overall, we did not find any support for our predicted associations between personality and DTC. However, our analysis showed that our failure to support this hypothesis could be due to the moderating effect of gender on these associations. Indeed, when we tested, on an exploratory basis, whether these associations are gender-specific, a different and interesting picture emerged. While for women Conscientiousness was negatively associated with cognitive cost, for men Extraversion was positively associated with motor cost.

The first finding emerging from this study is that women with high Conscientiousness demonstrated a relatively lower cognitive cost during DT. The added cognitive task presented in the current study, the subtraction by 3, is considered a relatively highly complex task [35]. The way in which older adults divide their attention during this task combination (walking with subtraction by 3) could be explained by either the limited resources model or by the task prioritization model [8]. Several personal factors may determine the way in which people divide their attention between two tasks: hazard estimation, postural reserve, level of familiarity with the task, mood and character. Participants with postural reserve may direct their attention to the cognitive task [43]. In line with the literature on personality and health, it is reasonable to assume that women with high Conscientiousness directed their attention more to the cognitive task and had relatively low cognitive cost due to good performance. High Conscientiousness was previously linked to better health outcomes such as increased gait speed and reduced risk for disability and mobility disorders [20, 26, 28]. Additionally, This explanation is in agreement with that of previous studies demonstrating an association between Conscientiousness and executive function [44], which is supported by neuro-imaging studies [45]. Optimal executive function is one of the key determinants of effective resource allocation and is strongly associated with a decreased risk of falls in the elderly [7].

A second relationship demonstrates that for men Extraversion is positively associated with motor cost. This finding contradicts recent studies showing that low Extraversion is associated with low ability to divide attention during DT walking in older adults [29]. Results suggest that in men with higher levels of Extraversion, the cost of performing the walking task in DT compared with ST is higher than in those with lower Extraversion. This finding could indicates a relatively unsafe walking strategy among extraverted men and ineffective ways of dividing attention. Extroversion encompasses the tendency toward positive mood, sociability, and activity [46]. The tendency to be friendly toward others and to generally have positive emotions and attitudes may indicate a predisposition to engage in a broad range of social behaviors, which include an active, busy, or engaged lifestyle that promotes better physical [47] and cognitive health [48]. These findings may indicate that men with higher extroversion may pay more attention to the cognitive task while walking. However, this finding is not consistent with previous studies that demonstrated an association between high levels of Extroversion and reduced risk for disability [28].

We may speculate that different social roles and challenges for men and women [49] account for these results. As indicated above, the two FFM factors of Extraversion and Conscientiousness have consistently been found to be significant predictors of reduced risk of disability with aging [28], but almost no past study has tested the possibility that these effects differ by gender. Future research might address this issue. The findings reported here should be interpreted in light of our study’s potential limitations. The study population comprised relatively high-functioning individuals, and the sample was relatively small. It is possible that variables not included in the study may better illuminate the association between personality traits and gait with DT. These variables could include in-home mobility monitoring with sensors, which might explain how daily routines affect personality and lead to better outcomes while walking. Finally, the cross-sectional design limited our ability to evaluate the relationship between these variables over time.

Additional research is needed to clarify the role of objective daily functioning in this relationship, and to discover other pathways to a better understanding of these relationships. In addition, a longitudinal study design should be used to evaluate these relationships over time. This study adds important information to the understanding of mobility deterioration in older adults. Understanding the contribution of personality to mobility deterioration may lead to early detection of people at risk of falls, as well as to the development of personality-tailored interventions to prevent mobility decline in the aging population.

## Conclusions

Findings from this study highlight the association between personality and DT performance during walking in older adults. Specifically, they suggest that Conscientiousness and Extroversion are associated with better and safer functioning in older adults in gender-specific ways. More specifically, future researchers testing the association between personality traits and DTC should formulate gender-specific hypotheses.

## Abbreviations

DT: Dual-task
DTC: Dual-task cost
FFM: Five-factor model
MC: Minimal cognitive impairment
MoCA: Montreal Cognitive Assessment
OLS: Ordinary least squares
ST: Single-task
